# Uniparental genetic markers to investigate hybridization in wild-born marmosets with a mixed phenotype among *Callithrix aurita* and invasive species

**DOI:** 10.1038/s41598-021-04276-7

**Published:** 2022-01-27

**Authors:** Denise Monnerat Nogueira, Rodrigo Salles de Carvalho, Andréa Maria de Oliveira, Thiago Silva de Paula, Daniel Gomes Pereira, Alcides Pissinatti, Silvia de Oliveira Loiola, Elizeu Fagundes Carvalho, Dayse Aparecida Silva, Helena Godoy Bergallo, Ana Maria dos Reis Ferreira

**Affiliations:** 1 Departamento de Genética, Instituto de Ciências Biológicas e da Saúde, Universidade Federal Rural do Rio de Janeiro, BR-465, Km 07, Seropédica, Rio de Janeiro, CEP 23897000 Brazil; 2 Programa de Educação Ambiental (PREA), Rio de Janeiro, Brazil; 3 Departamento de Genética, Instituto de Biologia Roberto Alcantara Gomes, Universidade do Estado do Rio de Janeiro, Rio de Janeiro, RJ Brazil; 4 Laboratório de Primatologia, Universidade Santa Úrsula, Rio de Janeiro, Brazil; 5 Centro de Primatologia do Rio de Janeiro, INEA, Guapimirim, Rio de Janeiro, Brazil; 6 Laboratório de Diagnósticos por DNA, Universidade do Estado do Rio de Janeiro, Rio de Janeiro, Brazil; 7 Departamento de Ecologia, Instituto de Biologia Roberto Alcantara Gomes, Universidade do Estado do Rio de Janeiro, Rio de Janeiro, Brazil; 8 Departamento de Patologia e Clínica Veterinária, Universidade Federal Fluminense, Rio de Janeiro, Brazil

**Keywords:** Invasive species, Molecular ecology

## Abstract

The native marmoset of the Southeastern Atlantic Forest in Brazil is among the 25 most endangered primates of the world. Hybridization with alien species is one of its main threats registered since the early 2000s based on phenotype, so far, without genetic confirmation. Using uniparental molecular markers, we analyzed 18 putative hybrids, captured from 2004 to 2013 in different localities of the Atlantic Forest. A nine base pair deletion in the *SRY* gene of *C. aurita* was used to investigate paternal ancestry. Maternal ancestry was assessed by DNA sequencing of ca. 455 bp from the *COX2* gene. Hybridization was confirmed for 16 out of the 18 marmosets since they inherited *COX2* haplotypes of the alien *C. penicillata* or *C. jacchus* and the *SRY* deletion specific to *C. aurita*. Two individuals inherited both parental lineages of *C. aurita*, which is probably related to backcrossing or hybrid interbreeding. The direction of hybridization of females with the matrilineal lineage of invasive species with males descending from the native lineage was predominant in our sampling. This is the first time that hybridization between *C. aurita* and invasive species has been confirmed through genetic analysis.

## Introduction

The buffy-tufted-ear marmoset, *Callithrix aurita* is endemic to the Brazilian Atlantic Forest, occurring in the states of Rio de Janeiro, eastern and northeastern São Paulo and adjacent parts of Minas Gerais. These areas, together with the states of Espírito Santo and southern Bahia, have long been recognized as a ‘core area’ for primates^1^, since they host 19 of the 22 species found in the Atlantic rainforest biome, being the number of species updated^2^.

The small distribution range, habitat destruction and in-situ hybridization with invasive species are the major threats to the conservation of *C. aurita*, however, the extent of the latter remains obscure^3,4^. The species once considered extinct in Rio de Janeiro state during the 1980s^1,5,6^ is currently among the 25 most endangered primates of the world^7^. It is also configured in the national and international lists of threatened species^4,8,9^ as endangered.

The *Callithrix* genus comprises six taxa, originally parapatric^10^. Among them, two species with the most extensive distribution, the common marmoset *C. jacchus* and the black-tufted-ear marmoset *C. penicillata*, emerged as invaders to the southeastern and southern regions of Brazil^1,11–14^. *Callithrix jacchus* is native to Bahia, Tocantins, Maranhão, Piauí, Ceará and Rio Grande do Norte, while *C. penicillata* is original to Central Brazil in the states of Mato Grosso do Sul, Goiás, Tocantins, Bahia, part of São Paulo and part of Minas Gerais^10^. In contrast to *C. aurita*, both species are generalists and inhabitants of secondary forests, which putatively enable them to have a greater degree of environmental plasticity to adapt to degraded areas and expand their territories^15,16^. These two species hybridize naturally in a contact zone of their original range in the northeast and by human-mediated introduction in Rio de Janeiro state^13^. There are no natural hybrid zones registered between *C. aurita* and the invasive species^5^.

Interspecific hybridization occurs when parental individuals are from genetically distinct populations, subspecies or species^17^. At the species taxonomic level, it results in progeny that carry a mixture of previously isolated gene pools. It occurs naturally at distribution boundaries between closely related species in hybrid zones that act as a "filter testing new genotypic combinations through natural selection”^18^. Thus, gene introgression may or may not occur and is considered a dynamic evolutionary mechanism capable of contributing to the genetic enrichment of species while preserving distinct biological entities^19^. New genotypic combinations can also allow for the exploration of new niches resulting in speciation. It is estimated to occur naturally in more than 10% of primate species^18^.

However, human accidentally induced hybridization has been a concern in conservation. The direct or indirect introduction of nonnative taxa by alteration of the habitat allows the expansion of territories of allopatric or parapatric species, and sometimes the suppression of reproductive isolation between sympatric species facilitates interbreeding^20^. Hybridization can be especially problematic for rare species contacting more abundant species^17^. Among the most relevant outcomes of hybridization is genetic introgression, i.e. the movement of a gene from one species to the genetic pool of another through repeated backcrosses between a hybrid and its original parent generation^17^. If hybridization events are rare, introgression may not occur since F1 individuals are subject to the same selective forces as others or even when common, because the offspring are sterile or less adapted^21^. The consequence may also be less severe for the rare species if there are pure stocks in isolated populations not affected by hybridization. If introgression is widespread over the entire distribution of the species, the outcome in general is the disintegration of the genome of the rare species in hybrid swarms and its assimilation by the most abundant species^21^.

According to distribution modeling, *C. penicillata* is the species with the highest potential of impact over *C. aurita* due to similarities in the pattern of environmental fitness between both species. Areas with more pronounced seasonality, higher altitudes and mild temperatures that adjust to *C. penicillata* are less suitable for the dispersion of *C. jacchus*^22^*.* However, this scenario can be changed because of global warming, where an expansion of the distribution of *C. jacchus* is predicted and the opposite for *C. penicillata* and *C. aurita*^23^.

Illegal pet trafficking is considered the main factor in the introduction of exotic *Callithrix* species in the southeastern region^12,24^, with habitat invasion probably favored by long-term Atlantic Forest disturbance^25^. The loss of biodiversity, both by deforestation and by climate change, reduces habitat heterogeneity, relaxes divergent selection, and promotes interspecific hybridization^26^.

Hybridization has been considered a threat to *C. aurita* since 2008^3^ when the first putative hybrids were described in the Serra dos Órgãos National Park (Parque Nacional da Serra dos Órgãos-PARNASO) municipality of Teresópolis in Rio de Janeiro state^27^, based on morphological features, followed by other publications based on the same grounds^27–29^. The appearance of the ear tufts, the pattern of the face and the coat coloration of the back are the main phenotypic features considered as a diagnosis for the three species in question, and thus of hybridization among them. Accordingly, with some authors, observed hybrids presented an intermediate phenotype to that of the parental species^28–32^. Vocalization is also intermediate between the pattern of the parental species^33^.

However, the detection of hybrids only assuming that they will be phenotypically intermediate to parental species can represent an inaccuracy, considering that there is greater morphological variation within and among populations than is recognized and, in general, the hybrids can express a mosaic of parental phenotypes^34^. For example, an unexpected finding was registered in four putative male hybrids with an intermediate phenotype between *C. jacchus* and *C. penicillata,* since they carried an acrocentric Y chromosome described only for *C. aurita*^35^. Once F1 has been generated and if the hybrids are viable and fertile, the backcross with one of the parental species is facilitated, making it impossible to distinguish them phenotypically from the parental species^36^. The outcome is that the frequency of backcrosses in nature is probably underestimated.

A factor that can contribute for the variety of phenotypes in marmoset hybrids is the successful interbreeding, probably due to the same diploid number, 2n = 46, and the morphological similarity among the autosomes of five *Callithrix* species (*C. flaviceps* was not studied). The five studied species differ only in terms of a small polymorphic uni- or biarmed Y chromosome and its Ag-NOR banding pattern^35,37,38^. Ex situ hybridization experiments during the 1970s and the beginning of the 1980s proved a degree of fertility of the hybrids among the six species, but with apparent reduced fertility in hybrids between *C. aurita* and *C. jacchus*^5^. Once fertile, backcrossing to one or both parental taxa, as well as mating among the hybrids, can generate hybrid swarms^21^.

Interspecific breeding has been suggested by cytogenetic analyses based on the finding of a small acrocentric Y chromosome exclusive to *C. aurita* in three male marmosets with a mixed phenotype^31,39^. Additionally, by DNA sequencing of the cytochrome c oxidase subunit II gene (*COX2*), the matrilineal lineage of *C. aurita* was identified in hybrids in whose group the dominant male was *C. aurita* with a female descendant of *C. jacchus*^30^. Nevertheless, neither record genetically confirmed hybridization among the native species and the invasive species.

A genetic polymorphism that can distinguish a male of *C. aurita* from the other marmosets is a deletion of nine base pairs (bp) in the *SRY* (sex-determining region of the Y chromosome) gene sequence, which is found exclusively in this species^40^. This polymorphism can be useful to determine whether the patrilineal lineage of the free-living putative hybrids comes from native or invasive species. In turn, specific haplotypes of *COX2* can represent a molecular marker^30^ to identify the matrilineal lineage.

Our main goal was to investigate the occurrence of in situ hybridization using uniparental markers amongst a sample of marmosets with a mixed phenotype, captured over nine years from different places of the Atlantic Forest and to assess whether there is a pattern in mating.

## Results

### Sample data

**Figure 1:**
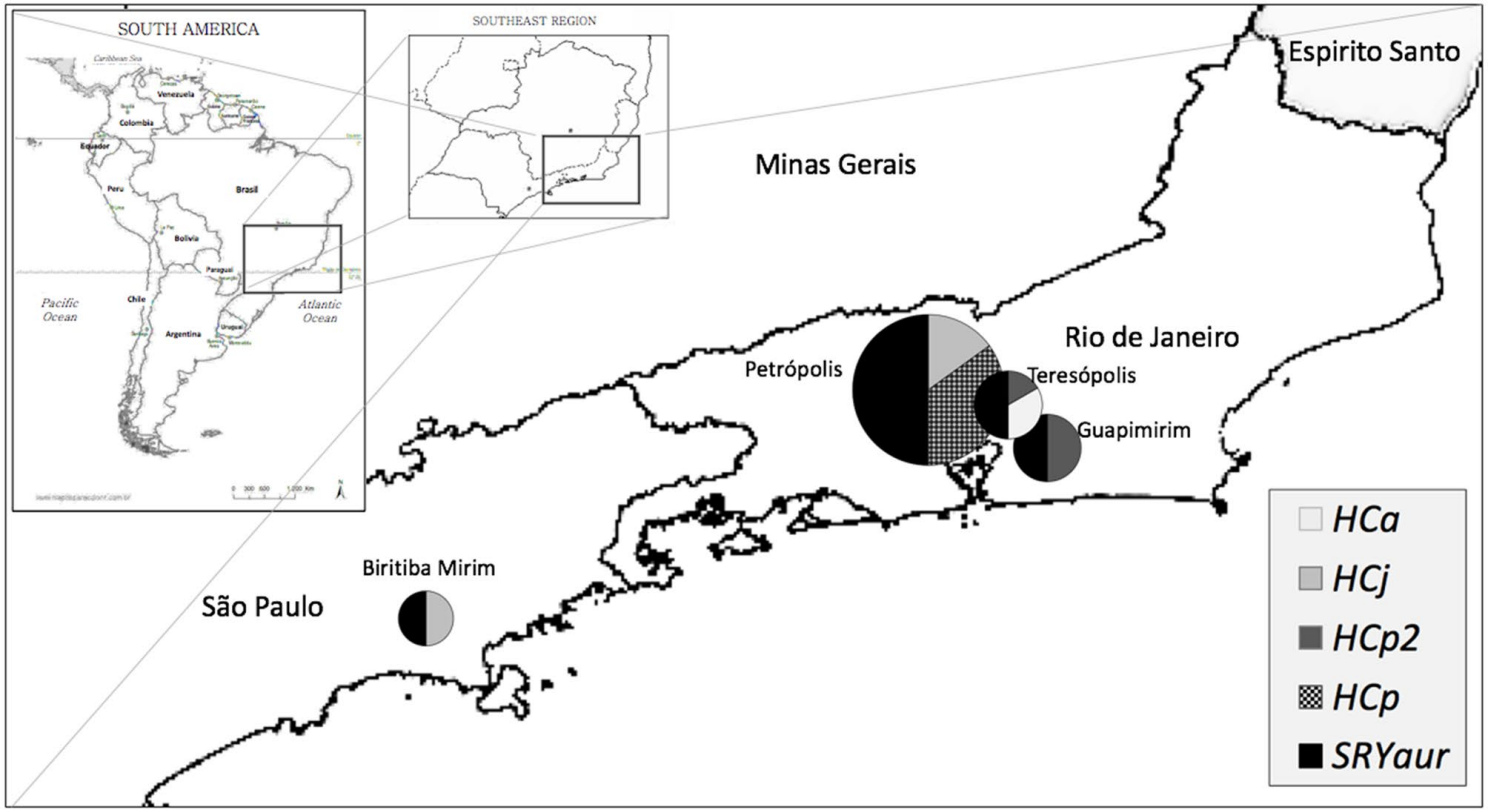
Place of origin and sampling of the 18 individuals of *Callithrix* sp. studied. (**A**). Map of South America; (**B**). Southeastern Brazil; (**C**). Sampling locations at Rio de Janeiro state: Guapimirim, Teresópolis and Petrópolis. In São Paulo state, only Biritiba Mirim. The size of the circles represents the sample number. Half of the circle in black represents the *SRY* haplotype with the deletion of nine base pairs exclusive to *C. aurita* (*SRYaur*), identified in all hybrids. In the other half, the *COX2* haplotypes identified among the samples are represented. Two individuals from Guapimirim carried the haplotype of *C. aurita* (H*Ca*). In all the others, the haplotypes of invasive *C. jacchus* (H*Cj*) and *C. penicillata* (H*Cp* and H*Cp*2) were identified. (Map created by R. S. Carvalho using QGIS 3.16. QGIS Geographic Information System. QGIS Association. http://www.qgis.org).

Eighteen marmosets considered putative *C. aurita* hybrids (*Csp*) were captured in three different localities in the state of Rio de Janeiro and one place in the state of São Paulo, Brazil, from 2004 to 2013 (Fig. 1, Table 1).

**Table 1:** Data concerning 18 *Callithrix* sp. (*Csp*1-18) and the positive controls sampled: *C. aurita* (*Ca*1-6), *C. jacchus* (*Cj*1-5) and *C. penicillata* (*Cp*1-4). For each sample, the electropherogram of the fragment of the *SRY* gene and the *COX2* haplotype amplified (patrilineal and matrilineal lineage, respectively) is shown.

ID (Sex)	Geographic Origin/State	Coordinates	Birth/Year of Capture	*SRY*	*COX2*
*Ca*1 (M)	Biritiba Mirim, SP	23° 28′ S/46° 11′ W	2003		H*Ca*
*Ca*2 (F)	PARNASO, RJ	22° 27′ S/42° 57′ W	2013		H*Ca*
*Ca*3, *Ca*4(M)	PARNASO, RJ	22° 27′ S/42° 57′ W	2013		H*Ca*
*Ca*5, *Ca*6 (M)	Natividade, RJ	21° 01′ S/41°59′ W	2015		H*Ca*
*Cj*1 (M)	CPRJ	22° 29′ S/42° 54′ W	2009		H*Cj*
*Cj*2 (M)	Bosque da Barra, RJ	23° 00′ S/43° 24′ W	2011		H*Cj*
*Cj*3 (M)	Nogueira, RJ	22° 24′ S/43° 07′ W	2012		H*Cj*
*Cj*4 (F)	Recife, PE	8° 2′ S/34° 53′ W	2014		H*Cj*
*Cj*5 (M)	Recife, PE	8° 2′ S/34° 53′ W	2014		H*Cj*
*Cp*1 (F)	Correias, RJ	22° 26′ S/43° 08′ W	2012		H*Cp*
*Cp*2 (M)	Correias, RJ	22° 24′ S/43° 07′ W	2012		H*Cp*
*Cp*3 (F)	Juiz de Fora, MG	21° 45′ S/43° 16′ W	2012		H*Cp*
*Cp*4 (M)	Brasília, DF	15° 44′ S/47° 44′ W	2014		H*Cp*
*Csp*1 (F)	Biritiba Mirim, SP	23° 28′ S/46° 11′ W	2004		H*Cj*
*Csp*2 (M)	Biritiba Mirim, SP	23° 28′ S/46° 11′ W	2004		H*Cj*
*Csp*3, *Csp*4 *Csp*5 (M)	Guapimirim, RJ	22° 32′ S/42° 58′ W	2005		H*Cp*2
*Csp*6 (M)	PARNASO, RJ	22° 27′ S/42° 57′ W	2005		H*Ca*
*Csp*7 (M)	PARNASO, RJ	22° 27′ S/42° 57′ W	2005		H*Cp*2
*Csp*8 (M)	PARNASO, RJ	22° 27′ S/42° 57′ W	2008		H*Ca*
*Csp*9, *Csp*10 (M)	Nogueira, RJ	22° 24′ S/43° 07′ W	2012		H*Cj*
*Csp*11 (F)	Nogueira, RJ	22° 24′ S/43° 07′ W	2012		H*Cj*
*Csp*12, *Csp*13, *Csp*14, *Csp*15, *Csp*16, *Csp*17 (M)	Nogueira, RJ	22° 24′ S/43° 07′ W	2013		H*Cp*
*Csp*18 (F)	Nogueira, RJ	22° 24′ S/43° 07′ W	2013		H*Cp*

**ID-** sample identification; **M**- Male; **F**- Female; **H*Ca***- *COX2* haplotype of *C. aurita*; **H*Cj***- *COX2* haplotype of *C. jacchus*; **H*Cp*** and **H*Cp2***- *COX2* haplotype of *C. penicillata;* **del**- *SRY* deletion of nine base pairs with an electropherogram peak of 196 base pairs (bp); **ins**- *SRY* insertion of nine base pairs with an electropherogram peak of 205 bp; **RJ**- Rio de Janeiro state; **SP**- São Paulo state; **PARNASO**- Serra dos Órgãos National Park, Teresópolis, RJ, Brazil.

Among the putative hybrids, there were 15 males and three females. Fifteen wild-born individuals of *Callithrix aurita* (six), *C. jacchus* (five) and *C. penicillata* (four) were sampled as positive controls (Table 1).

**Figure 2:**
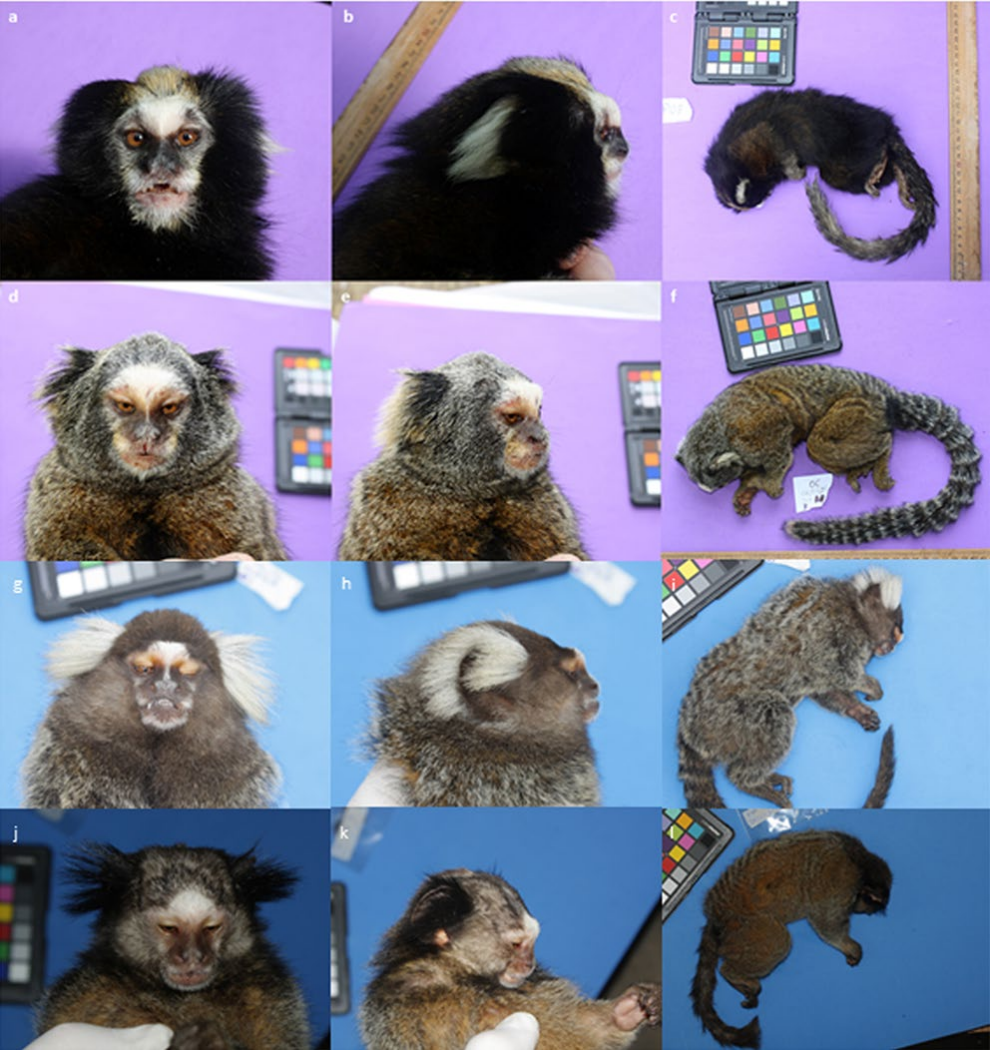
*Callithrix aurita* (above) and an example of hybrid phenotypes (below) of the sampled individuals of *Callithrix sp.*: (**a**, **d**) loss of the yellowish supracranial line peculiar of *C. aurita*; (**b**, **e**) short ear tufts, in some cases fan-shaped with darker color; **c**, **f**) lighter color of the body with eventual grooves; (**g**–**i**) *C. jacchus* phenotype; (**j**–**l**) *C. penicillata* phenotype (Photographs by R. S. Carvalho).

In general, the putative hybrids exhibited short ear tufts, in some cases fan-shaped with darker color. Commonly, the loss of the yellowish supra-cranial line peculiar to *C. aurita* was observed. The color of the body was lighter with eventual grooves (Fig. 2). These features confer to the individuals sampled a mixed pelage pattern of the tufts and the back, suggesting that they are hybrids between *C. aurita* and *C. penicillata* or *C. jacchus*^29,32,41^ (since both alien species are observed at the sampling locations).

### SRY analysis

**Figure 3:**
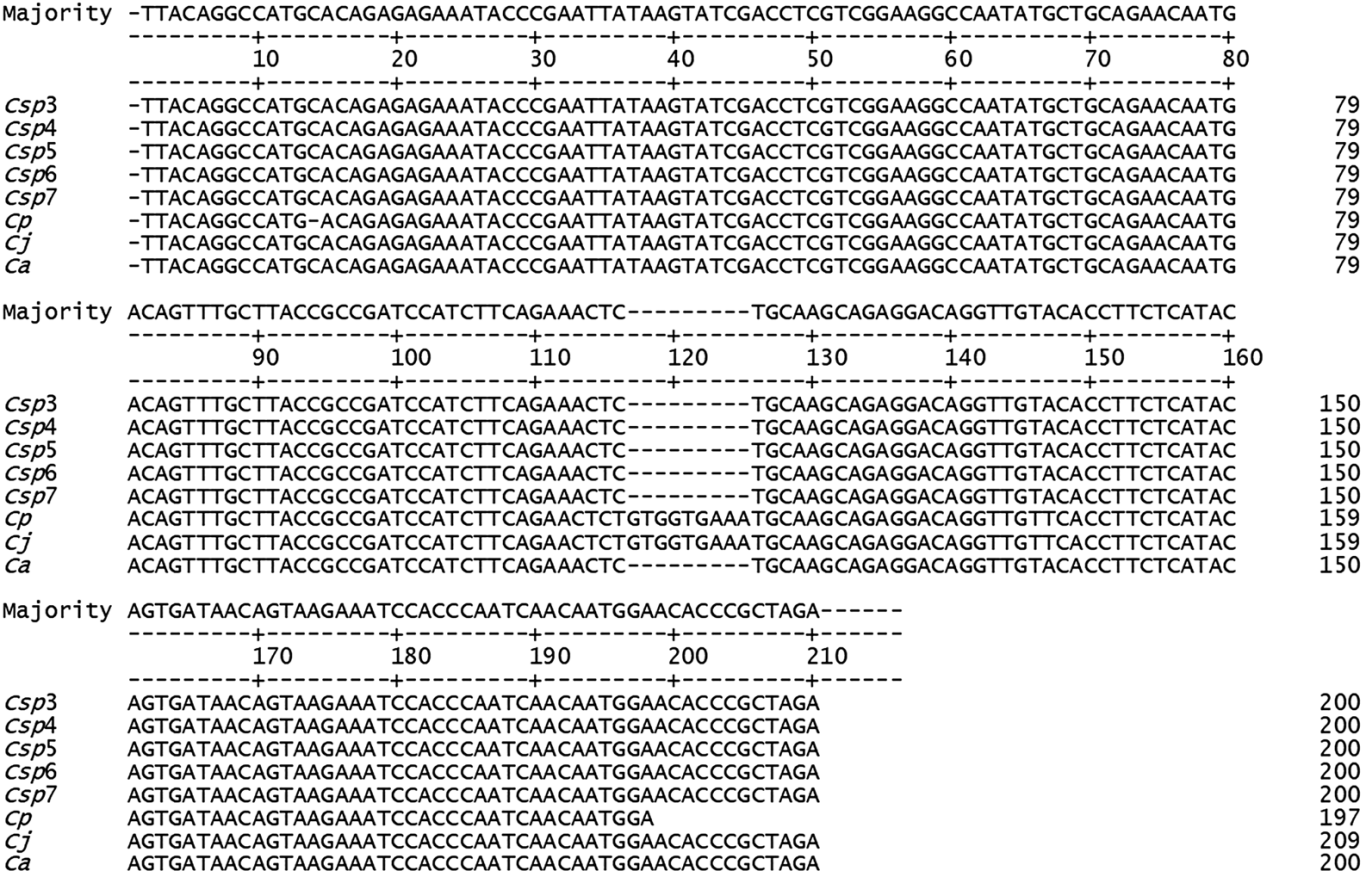
Alignment sequence of 210 base pairs (bp) of the *SRY* (sex-determining region Y) gene in five *Callithrix* sp. males (*Csp*3, *Csp*4, *Csp*5, *Csp*6 and *Csp*7) and one male of each *C. penicillata* (*Cp*), *C. jacchus* (*Cj*) and *C. aurita* (*Ca*). The dashes indicate the nine base pair deletion 117_125delTAAGTATCG observed in *C. aurita* and in the five *Callithrix* sp.

In our preliminary analysis to test the efficacy of the SRY primer set developed in this research to ascertain the patrilineal lineage of the putative hybrids, we sequenced the PCR products of one pure individual of each *C. aurita* (*Ca*), *C. jacchus* (*Cj*) and *C. penicillata* (*Cp*) used as a positive control and five putative hybrids of *Callithrix* sp. (*Csp*3, *Csp*4, *Csp*5, *Csp*6 and *Csp*7). We obtained the *SRY* nine base pair deletion 117_125delTAAGTATCG exclusive to *C. aurita* in the *Ca* positive control and in the five *Csp* samples tested. At the same position is the insertion in the *Cj* and *Cp* positive controls, which presented identical DNA sequences (Fig. 3).

Afterward, using the same primer set labelled with 6-FAM fluorescent dye, an amplicon of the *SRY* of 196 bp was observed for all six *Ca* positive controls and 18 putative hybrids samples attesting to the patrilineal lineage of the native threatened species. For all nine positive controls of *Cj* and *Cp* an electropherogram peak of 205 bp was generated (Table 1 provides an example of the electropherogram for each subset of obtained peaks. The image of the 33 peaks can be found in Supplementary Fig. S1 and S2 online).

The amplification of the *SRY* fragment in the eight female samples among the positive controls (*Ca*2, *Cj*3, *Cj*4, *Cp*1 and *Cp*3) and the putative hybrids (*Csp*1, *Csp*11, and *Csp*18) was possible due to the occurrence of placental anastomosis which allows hematopoietic chimerism resulting from the high incidence of twin gestation in callitrichids^42^.

### *COX2* analysis

**Figure 4:**
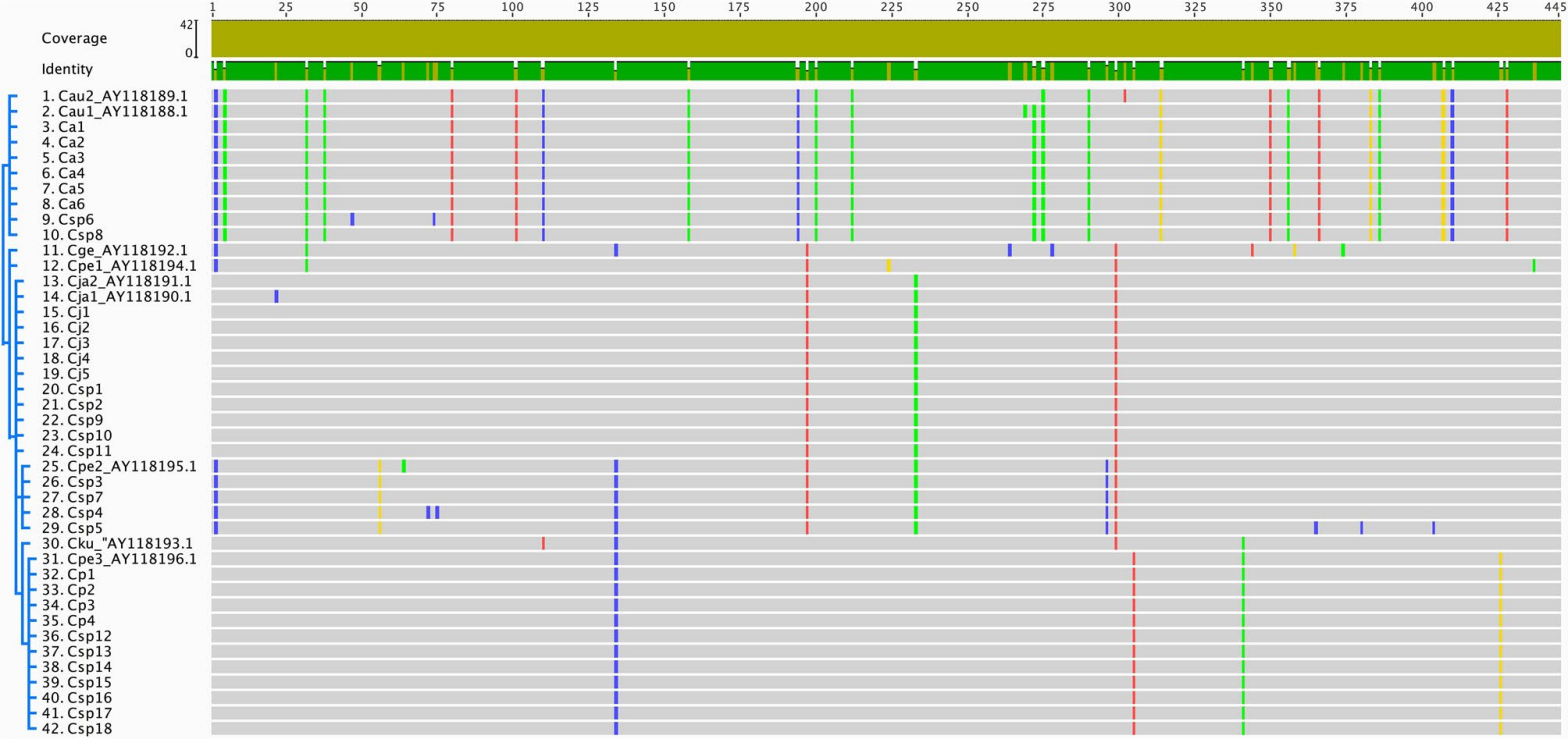
Alignment of the DNA sequencing of 445 base pairs of the cytochrome c oxidase subunit II (*COX2*) gene. One haplotype was detected for *Callithrix aurita* (*Ca*), one for *C. jacchus* (*Cj*), one for *C. geoffroyi,* one for *C. kuhlii* and three for *C. penicillata* (*Cp*). The 18 sequences from the *Callitrix* sp. (*Csp*) free-living individuals aligned with *Ca*, *Cj*, and two *Cp* haplotypes. The gray color represents similarities among the nucleotide sequences of all individuals. The blue colored bar represents cytosine in that position, yellow for guanine, green for thymine and red for adenine. The sequences identified by AY were obtained from GenBank®.

**Figure 5:**
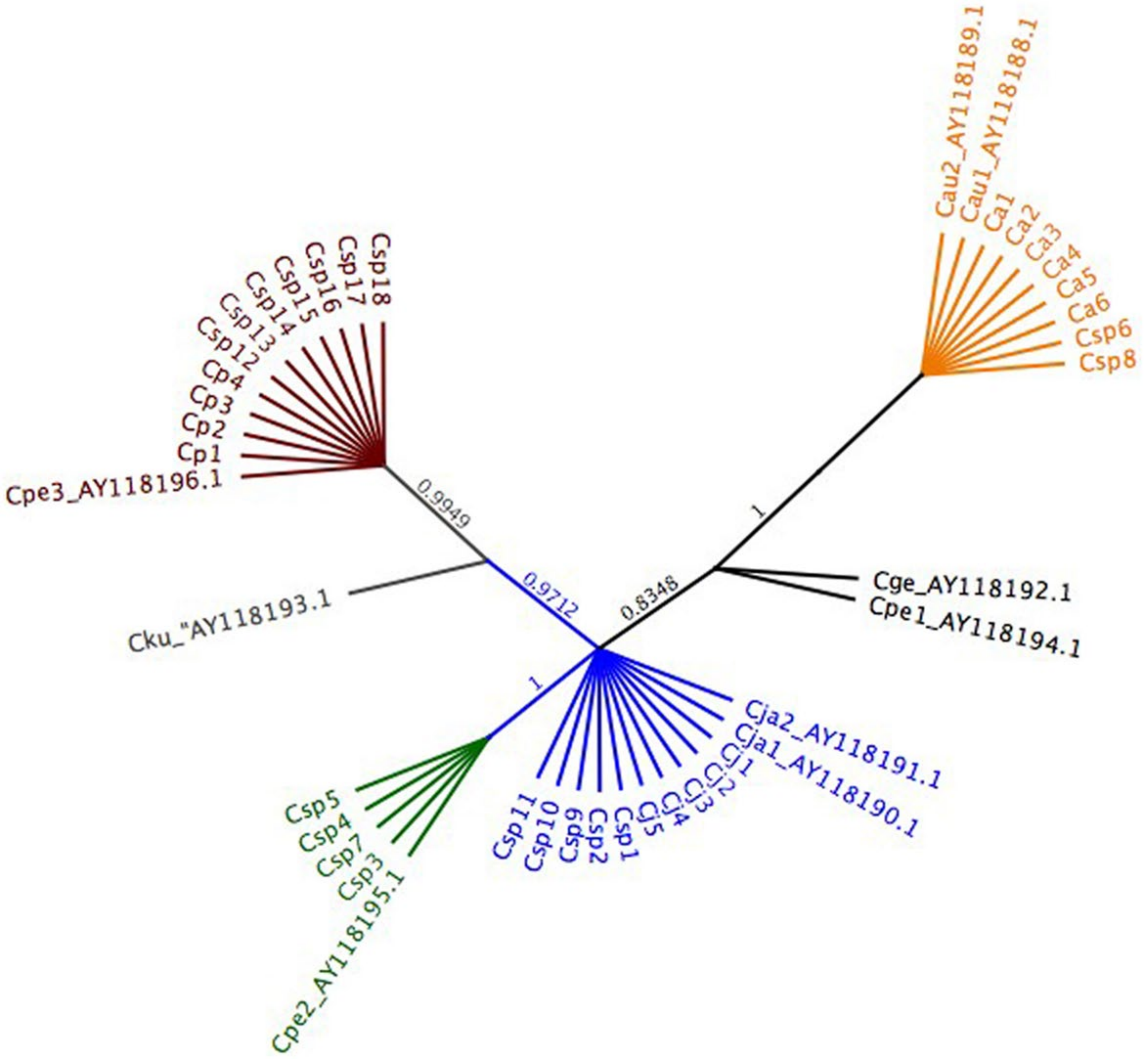
Phylogenetic Bayesian tree was built with MRBAYES 3.2.1 based on alignment of 445 base pairs of the cytochrome c oxidase subunit II gene alignment from *Callithrix aurita* (Ca, orange), *C. jacchus* (Cj, blue), and *C. penicillata* (Cp 1–3, brown, green and black). Sequences of *C. kuhli* (Cku) and *C. geoffroyi* (Cge) were also included. The 18 *Callithrix* sp. (Csp) clustered with Ca, Cj, Cp and Cp2 haplotypes. GenBank® sequences are designated by AY. Numbers on branches indicate posterior probabilities.

PCRs with the COX2 primer set amplified a single product of the expected size of ca. 445 bp for all the *Callithrix* positive controls and for the 18 *Csp* samples analyzed. Through the alignment of DNA sequences of the positive controls and GenBank® sequences of each species, one *COX2* haplotype was found for *Callithrix jacchus* and *C. aurita*, identified as H*Cj* and H*Ca*, respectively (Fig. 4). Among the 18 sequences obtained from the putative hybrids we found five individuals with H*Cj* (*Csp*1, *Csp*2, *Csp*9, *Csp*10 and *Csp*11) and two with H*Ca* (*Csp*6 and *Csp*8). Regarding *C. penicillata*, three haplotypes were detected (Figs. 4 and 5). One of them corresponds to the GenBank® sequence Cpe1_AY118194.1, which clustered with the *C. geoffroyi* Cge_AY 118,192.1 haplotype and did not cluster with any of the *Callithrix* sp. neither with our positive control samples. The second haplotype identified as H*Cp* was shared by four of our positive controls, Cpe3_AY118196.1 from GenBank® and seven *Callithrix* sp. (Fig. 5). The third haplotype, H*Cp*2, was shared by Cpe2_AY118195.1, and four of our *Callithrix* sp. samples (Fig. 5). For *C. kuhli*, one *COX2* haplotype, AY118193.1, represented a specific branch (Fig. 5).

Each of the five haplotypes identified was separated by at least one variable of a total of 37 polymorphic sites. Haplotype diversity was 0.8205 ± 0,0356, and nucleotide diversity (π) was 0,0313 ± 0,0159. Among the haplotypes identified, there were 22 diagnostic variable sites for *C. aurita*, nine for *C. penicillata* and one for *C. jacchus* (see Supplementary Table S1 online). Most nucleotide changes among the GenBank® sequences of pure individuals and those mitochondrial haplotypes found in our positive controls and the hybrids are transitions that resulted in synonymous substitutions. Only four nonsynonymous substitutions were detected. One transition at *m.84T* > *C* specific to Cja1_AY118190.1 resulted in *p.L84P*, which clustered with another GenBank® sequence, those from our positive controls and five hybrids. Specific of Cpe2_AY118195.1, a transition *m.126C* > *T* resulted in the exchange of *p.T42M*. Four hybrids clustered with this haplotype identified as *HCp*2. Two transversions, one detected in all *C. aurita* samples *m.429A* > *G*, led to the replacement of *p.V143M* and the other *m.489G* > *A* changing *p.A165T* was present in Cp3_AY118196.1, where all our positive controls of *C. penicillata* and seven hybrids clustered, representing the H*Cp* haplotype (see the nucleotide and amino acid alignment in Supplementary Fig. S3a, S3b, S3c and S4 online).

The unrooted phylogenetic tree, generated by the alignment of the *COX2* sequences, including those from GenBank®, shows the greatest genetic variability of *C. penicillata* forming three well-defined clusters with posterior probabilities over 0.83 of node support (Fig. 5). One cluster is closer to that of *C. geoffroyi* (Cge_AY118119.1). Our *Cp* positive controls and seven *Csp* (12–18) clustered with Cpe3_AY118196. Four *Csp* (3–5 and 7) clustered with Cpe2_AY118.195.1, phylogenetically closer to *C. jacchus*. The sequences of none of the positive controls grouped with *C. geoffroyi* or *C. kuhlii*.

## Discussion

Hybridization was undoubtedly confirmed for 16 out of the 18 free-living marmosets studied since all of them inherited the *SRY* deletion 117_125delTAAGTATCG specific to *C. aurita* and *COX2* haplotypes of *C. penicillata* or *C. jacchus*. The molecular markers of patrilineal and matrilineal ancestry combined here were suitable to attest hybridization in almost 90% of the sampled individuals. Two male individuals, identified as *Csp*6 and *Csp*8, from PARNASO in the Teresópolis municipality of Rio de Janeiro state, although exhibiting a mixed phenotype, inherited both parental lineages exclusive to *C. aurita* which is probably related to backcrossing or hybrid interbreeding. These two individuals were members of a group of *C. aurita* and *C. penicillata*, where the only adult male belonged to the native species^27^.

The hybrids sampled were distributed throughout the original area of occurrence of *C. aurita*, with one locality in São Paulo state at Biritiba Mirim and three localities of Rio de Janeiro state, in the municipalities of Teresópolis, Guapimirim and Petrópolis. At these localities, *C. penicillata* is present in almost 60% of the interspecific breeding, *C. jacchus* in 30% and less than10% of backcrossing to *C. aurita* was registered. Despite being a small sampling by location, the greater participation of *C. penicillata* in hybridization with *C. aurita* corroborates the expectation due to the similarities in the pattern of environmental fitness between both species^22^ and also with the greatest ex situ reproductive success observed between these species when compared to *C. jacchus*^5^.

Although the fertility of the hybrids had already been confirmed experimentally through many directional matings through the six species of *Callithrix*^5^ and in situ among *C. penicillata* and *C. jacchus*^24^*,* nothing is known about the fertility of free-living hybrids of *C. aurita* and still less about the patterns of in situ mating selection. The divergence of the Atlantic Forest marmosets occurred lightly more than 5 Ma in the Pliocene–Pleistocene scenario^43–45^. In birds, a typical young pair of sister species has only 1–2 Ma, and fertile hybrids still occur when species have nearly 7–17 Ma. Complete hybrid unfeasibility occurs when species are separated by approximately 11–55 Ma^46^. Therefore, considering only the phylogenetic proximity among the marmoset species, several kinds of crossings could result in viable and fertile hybrids representing an additional competitor to the threatened *C. aurita*.

We drew attention to the fact that most individuals inherited the nuclear DNA marker of *C. aurita* and that the mitochondrial DNA marker of one of the invaders cannot ensure that they represent the F1 generation without previous life history knowledge. The same limitation exists to confirm backcrossing or interbreeding among hybrids as observed from samples of *Csp*6 and *Csp*8 individuals who presented patrilineal and matrilineal DNA sequences of *C. aurita* but with phenotypes that correspond neither to the native species nor to the invasive ones (see Supplementary Fig. S5 online). Multiple nuclear markers are needed to attest to which generation they belong to and for a more complete understanding of the extent of introgression. Microsatellite markers^47–49^ and a panel of SNPs developed from *C. jacchus* and *C. penicillata*^50^ are already available and should be tested on C*. aurita* for this purpose.

The suspicion of hybridization based on the morphology of the Y chromosome and mixed phenotype in five samples^39^ analyzed here (*Csp*3, *Csp*4, *Csp*5, *Csp*6 and *Csp*7) was now confirmed with the SRY and COX2 primer sets. Due to the subtle difference in the morphology among the Y chromosome of the native species and the invasive species and the polymorphism of this chromosome observed for the invasive species^35,38^, it is advisable to confirm through molecular genetics the inheritance of the *C. aurita* Y chromosome in hybrids based on cytogenetics^31^. Since *SRY* 117_125delTAAGTATCG is specific to *C. aurita*, the SRY primer set is suitable for this purpose.

According to *COX2*, we found greater genetic variability in *C. penicillata* than in *C. jacchus* and *C. aurita*. Our *C. penicillata* samples, engendered three haplotypes forming well-defined clusters with above 83% node support. Considering that Cge_AY118119.1 (representing *C. geoffroyi*) and Cpe1_AY118194.1 (representing *C. penicillata*) are from GenBank® and that there is no other sample matching them, we will not discuss their proximity. However, the Cpe2_AY118195.1 and Cpe3_AY118196.1 haplotypes, which matched our samples and generated two distinct groups, showed greater distances between each other than the one shown by the Cpe2_AY118195.1 and the *C. jacchus* cluster. This unprecedented result may suggest hidden phylogenetic diversity that may be revealed through extensive phenotype-genetic research along the *C. penicillata* home range.

Apparent directional mating was observed between males carrying the *C. aurita* Y chromosome with invasive females or at least female descendants of matrilineal lineage of the invaders. All 18 hybrids sampled over nine years in different places in the Atlantic Forest of RJ and SP carry the Y chromosome of *C. aurita* and 16, the *COX2* haplotype of *C. jacchus* or *C. penicillata*. The occurrence of the small isolated populations in contact with the closely related invasive species in a fragmented habitat is much more likely to hybridize because of the difficulty of finding mates of the same species^17^, the same scenario experienced by *C. aurita* in the Atlantic forest. The population decline and the consequent reduction in the number of native females for breeding may lead to hybridization as an alternative strategy to carry on reproduction. Considering that in most primates, females are philopatric and males disperse, male-mediated asymmetric introgression is the most likely outcome^18^. The prevalence of males of one population/species mating with heterospecific females, instead of reciprocal crosses, has already been observed for other mammalian taxa. For example, the introduced American mink (*Mustela vison*) is more numerous, and males are larger than the European threatened native mink (*Mustela lutreola*) which favors them to mate with European mink females^51^. As male of *C. aurita* is larger than the invaders, it could be one factor favoring their success with congeneric females. Added to the reduction of encounters, habitat devastation is recognized for favoring hybridization, since the modification of the environment can hinder communication and recognition of conspecifics^52^. Hybridization in São Paulo and Rio de Janeiro states represented in our sample can be boosted by deforestation, which increased just this year by up to 400% in São Paulo and more than doubled in Rio de Janeiro^53^. The Atlantic Forest retains only 12.4% of its original vegetation^54^, which is highly fragmented, and the largest fragments generally do not exceed 50 hectares^53^. It is urgent to seek information in the field on populations of *C. aurita* that inhabit more extensive and preserved forest fragments to ascertain whether the establishment of exotic species occurs and what kind of interaction takes place between them. The pure populations of the native species can also act as a source of parental native genome that can mitigate hybridization effects^19^. In the municipality of Sapucaia, Rio de Janeiro, one of the few records of the occurrence of populations of *C. aurita* was made without the presence of aliens in the vicinity^55^.

This is the first genetic confirmation that hybridization takes place among *C. aurita* and the invasive species *C. jacchus* and *C. penicillata* over time, although based on sampling in a small extension of the species distribution. The uniparental molecular markers used, despite the limitations that we have already noticed, allowed basic aspects of hybridization to be raised based on newly generated data.

In the end, we highlight the fact that there are possibly among the hybrids studied here, individuals that carry approximately 50% and maybe more of the genomic composition of the threatened native species *C. aurita*. This percentage is among what is currently recommended by several researchers in this field as a reasonable limit for protection^56,57^ representing an advance in relation to the minimum of 75% proposed in the intercross policy of 1996^57,58^. In Brazil, the recent creation of a captive breeding program^12^ can provide the necessary support to deepen the genetic, reproductive and physiological research regarding *C. aurita* hybrids. However, to a coherent hybrid policy, ecological features and historical process that resulted in admixture individuals should be incorporated^57^. As already highlighted, we must bear in mind that each case of hybridization is peculiar and to be effective, conservation general rules must be reassessed^21,57^. Considering that the southeastern region of Brazil presents different scenarios where habitat loss and forest fragmentation coexist with some remaining but significantly large and continuous Atlantic Rain forested areas, we emphasize that relative to the hybridization among *Callithrix aurita* and invasive marmosets, we must think about it regionally.

## Methods

### Sampling location

Sampling of the pure individuals for positive controls occurred in areas of the Cerrado, Caatinga and of the southeastern region of the Atlantic Forest, the natural areas of occurrence of *C. penicillata*, *C. jacchus* and *C. aurita,* respectively. The positive controls were represented by six individuals of *C. aurita*, five *C. jacchus* and four *C. penicillata*, among which there were five females. All of them were wild born out of one *C. jacchus* (*Cj*1) born in captivity at Centro de Primatologia do Estado do Rio de Janeiro/CPRJ in 2009 (microchip number 030000007075), whose parents were wild born. Two individuals of *C. jacchus* were in captivity at the time of sample collection at the Wildlife Screening Center (Centro de Triagem de Animais Silvestres/CETAS/IBAMA) in Recife city in the state of Pernambuco, Northeast Brazil (original habitat of this species) and one *C. penicillata* in CETAS/IBAMA of Brasília city in the Midwest region of Brazil (original habitat of this species). They are identified by the respective initials of the pure species (*Ca*, *Cj* and *Cp*) followed by an Arabic numeral (Table 1).

The putative hybrid marmosets were sampled in areas of the Atlantic Forest, a highly threatened ecosystem, classified as a global hotspot of biodiversity, due to its exceptional concentration of endemic species and loss of more than 90% of its primary vegetation^59^ and is the natural area of occurrence of *C. aurita*. The putative hybrids were identified with the acronym *Csp* (*Callitrix* sp.) followed by an Arabic numeral (Table 1).

Five males out of the eighteen putative hybrids studied here (*Csp*3, *Csp*4 and *Csp*5 from Guapimirim and *Csp*6 and *Csp*7 from PARNASO) have already been analyzed by cytogenetics and according to the morphology of their Y chromosome they were supposed to be hybrids fathered by a *C. aurita* male or a hybrid with the Y chromosome of this species^39^. According to the subtle difference among the morphology of the Y chromosome of the *Callithrix* species, it is difficult to attest patrilineal lineage solely based on cytogenetic data. For this reason, those samples were included for analysis using nuclear and mitochondrial markers.

The free-living animals studied were captured in Tomahawk traps, baited with fruits, and sedated with ketamine hydrochloride (10 mg/kg) via intramuscular injection for morphological evaluation and blood sampling.

All research conducted followed guidelines for the ethical treatment of nonhuman primates approved by the Ethical Commission of the Instituto de Biologia Roberto Alcantara Gomes of Universidade do Estado do Rio de Janeiro (UERJ) for the Care and Use of Experimental Animals and adhered to Brazilian legislation number CEUA035/2014. Research permits were approved by the Brazilian Ministry of Environment, MMA/IBAMA/SISBIO number 31570-5.

The study was carried out in compliance with the ARRIVE guidelines (https://arriveguidelines.org).

### SRY and COX2 analysis

To investigate the paternal lineage we used the sex-determining region of the Y chromosome (*SRY*) gene based on GenBank® sequences (AF338377, AF338381 and AF338379) of *Callithrix*^40^ to design consensus primers FSRY (5’-TACAGGCCATGCACAGAGAG-3’) and RSRY (5´-CTAGCGGGTGTTCCATTGTT-3’) for amplification through polymerase chain reaction (PCR), of an amplicon of ca. 200 bp, which includes the nine base pair deletion exclusive of *C. aurita*^40^. The organization of the mitogenome of the five *Callithrix* species was assumed to be free of NUMTS according to the high-quality base calls with no ambiguous nucleotides that could indicate a population of nuclear DNA of mitochondrial origin^50,60^.

To analyze the matrilineal lineage we used the primer set which flanks mitochondrial cytochrome c oxidase subunit II (*COX2*) gene following the advised PCR protocol^30^.

DNA was extracted from blood samples through phenol–chloroform protocol^61^. PCRs to amplify the *SRY* fragment contained 20–25 ng of DNA, 1–2 µM of each primer, 0.25–1 U of *Taq* DNA polymerase Platinum (Invitrogen®), 1X *Taq* buffer, 1.5 mM (for *SRY*)—2.5 mM (for *COX2*) MgCl_2_, 0.2 mM dNTPs and ultrapure water for a total reaction volume of 25 µL. The amplifications were carried out in a Veriti® 96-well Thermal cycler (Applied Biosystems, Co.) under the following conditions: a denaturation step of 94 °C for 5 min, followed by 30 cycles at 94 °C for 1 min, 55 °C for 1 min, 72 °C for 1 min, and a final extension step of 72 °C for 10 min.

PCR products were checked by electrophoresis in 2% agarose gel dyed with ethidium bromide (10 mg/ml). The amplicons of *SRY* and *COX2* were purified with Sephadex Resin and sequenced in an ABI PRISM 3500 (Applied Biosystem, Co).

Sequencing of the *SRY* amplicon was performed for three positive controls, one of each *Callithrix* species and five putative hybrids to check for the presence of the nine base pair insertions/deletions described in the pure species. Once it was confirmed, the forward primer was labelled with 6-FAM fluorescent dye, and all the PCR products were analysed in a ABI PRISM 3500 system (Applied Biosystem, Co) to identify the size of the amplicons using Geneious software. Our purpose was to make the analysis of putative hybrids more agile, eliminating the DNA sequencing step.

All samples were sequenced for the *COX2* amplicon. The DNA sequences generated were aligned with Clustal W using the Megalign DNASTAR/Lasergene program, Inc. The GenBank® sequences of *C. aurita* (AY118189.1; AY118188.1), *C. jacchus* (AY118190.1; AY118191.1), *C. penicillata* (AY118194.1; AY118195.1; AY118196.1), *C. geoffroyi* (AY118192.1) and *C. kuhlii* (AY118193.1) were aligned with the haplotypes identified here to verify nucleotide and amino acid similarities using MEGA-X^62^. DNAsp6^63^ was used to identify parsimony informative sites for each species and to estimate haplotype and nucleotide diversity. MRBAYES 3.2.1^64^ was used to build the Bayesian tree to analyze phylogenetic relationships under an HKY85 substitution model using a Metropolis-coupled, Markov Chain Monte Carlo (MCMC). Four chains were run simultaneously for 500,000 generations in two independent runs, sampling trees every 100 generations. A burn-in of 1000 trees was carried out for each independent run. A consensus tree with nodal posterior probability support was obtained for five *Callithrix* species and *Callithrix* sp. samples.

## Supplementary Information

Supplementary Figure S1.

Supplementary Figure S2.

Supplementary Figure S3a.

Supplementary Figure S3b.

Supplementary Figure S3c.

Supplementary Figure S3d.

Supplementary Figure S4.

Supplementary Figure S5.

Supplementary Table S1.
