# Supplementary figures and images for: Uniparental genetic markers to investigate hybridization in wild-born marmosets with a mixed phenotype among Callithrix aurita and invasive species
Source: Sci Rep. 2022 Jan 27;12:1487. doi: 10.1038/s41598-021-04276-7 (PMC8795268; doi:10.1038/s41598-021-04276-7)

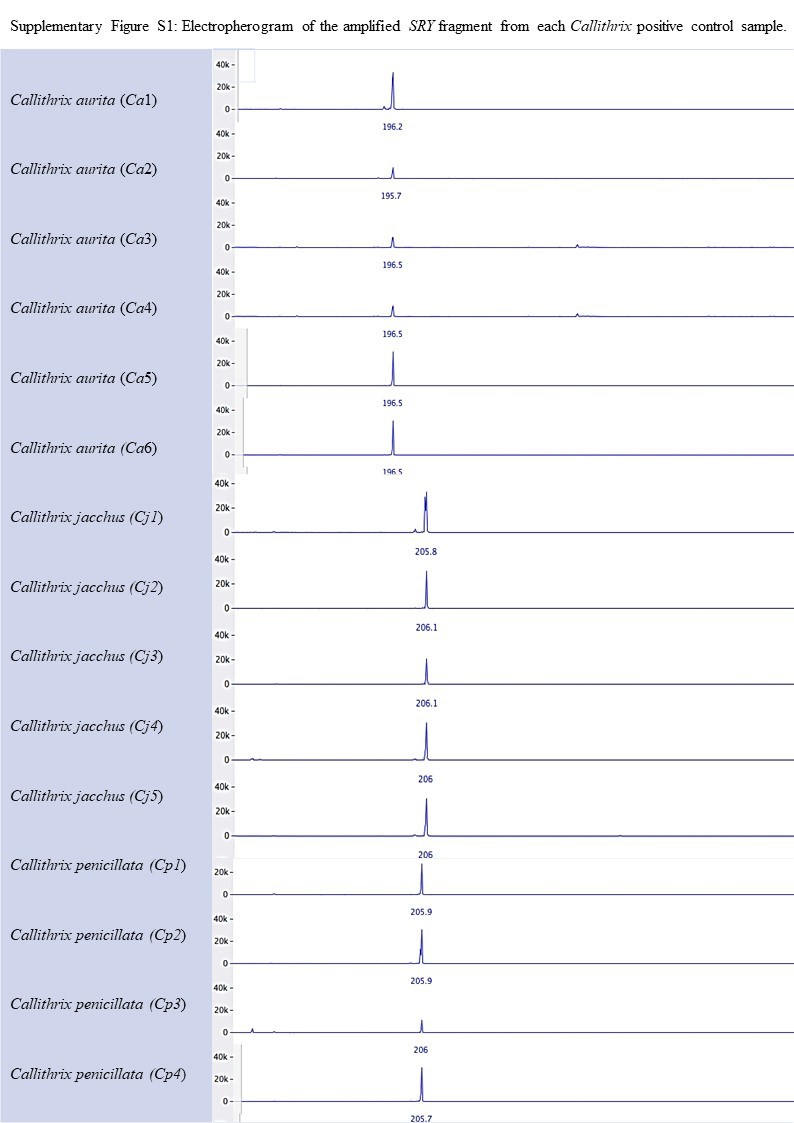

Supplement: Supplementary file 1 — Supplementary Figure S1. [file 41598_2021_4276_MOESM1_ESM.jpg]

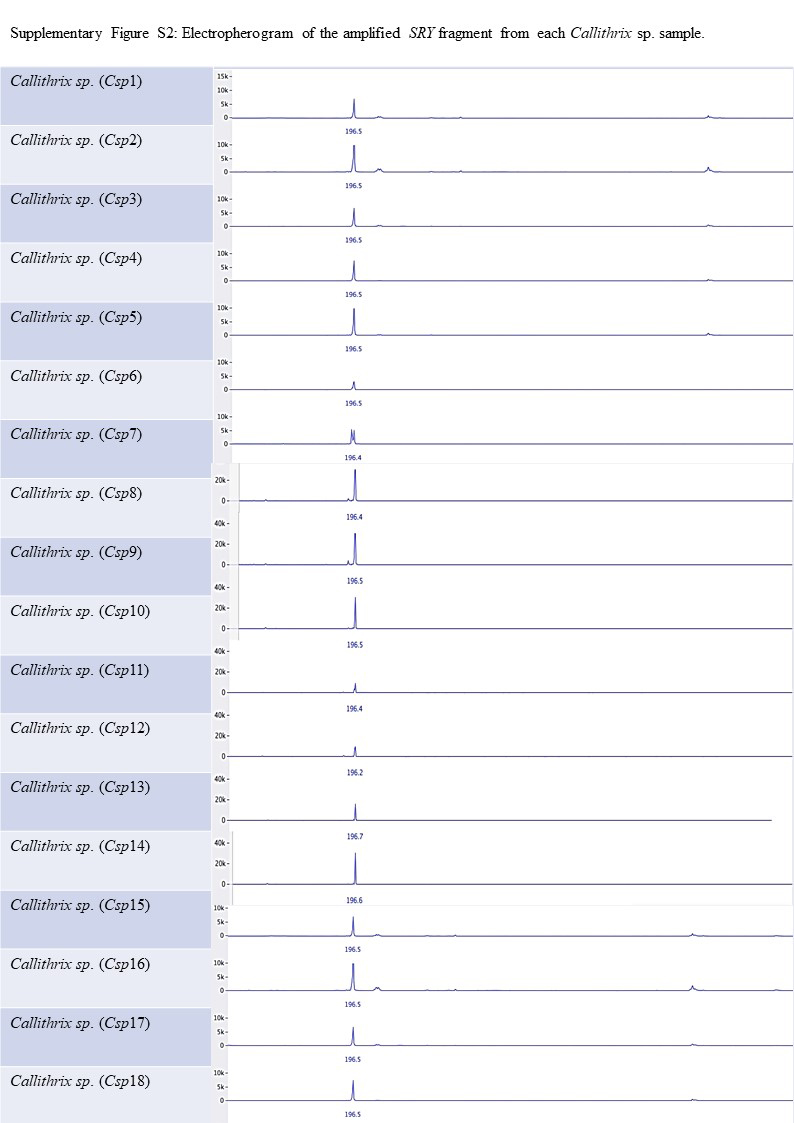

Supplement: Supplementary file 2 — Supplementary Figure S2. [file 41598_2021_4276_MOESM2_ESM.jpg]

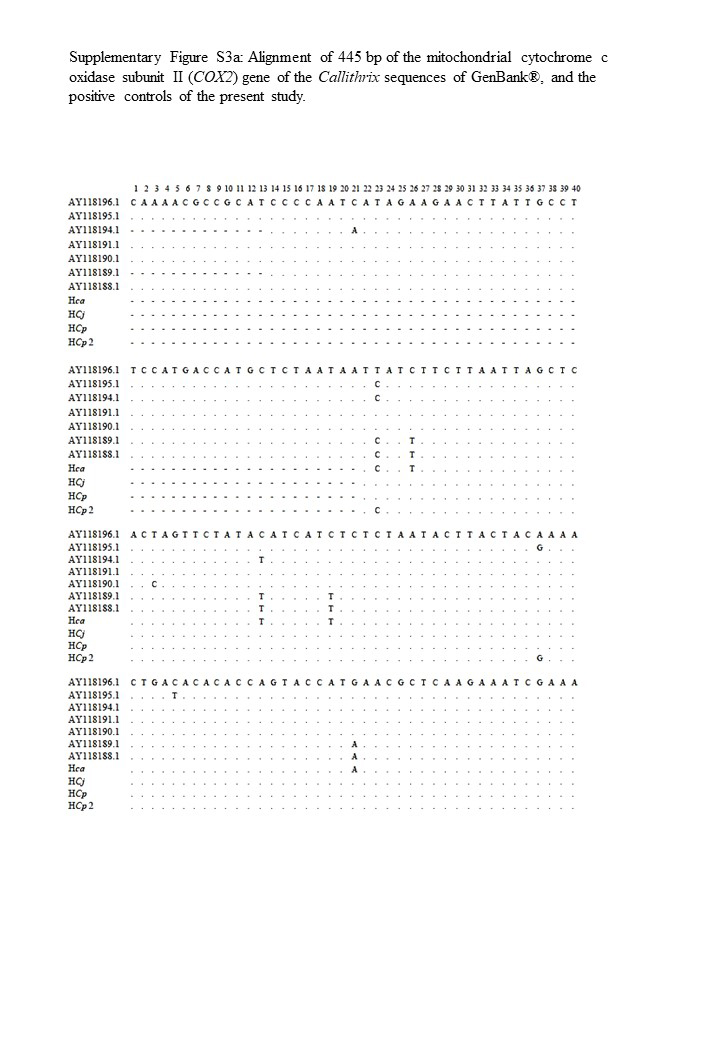

Supplement: Supplementary file 3 — Supplementary Figure S3a. [file 41598_2021_4276_MOESM3_ESM.jpg]

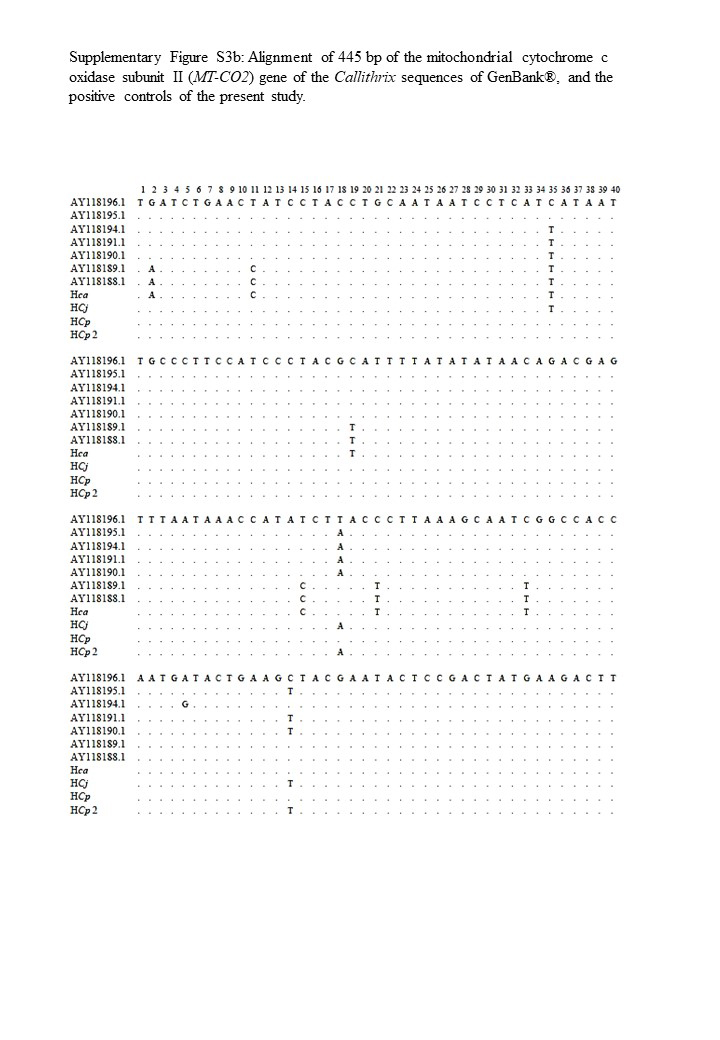

Supplement: Supplementary file 4 — Supplementary Figure S3b. [file 41598_2021_4276_MOESM4_ESM.jpg]

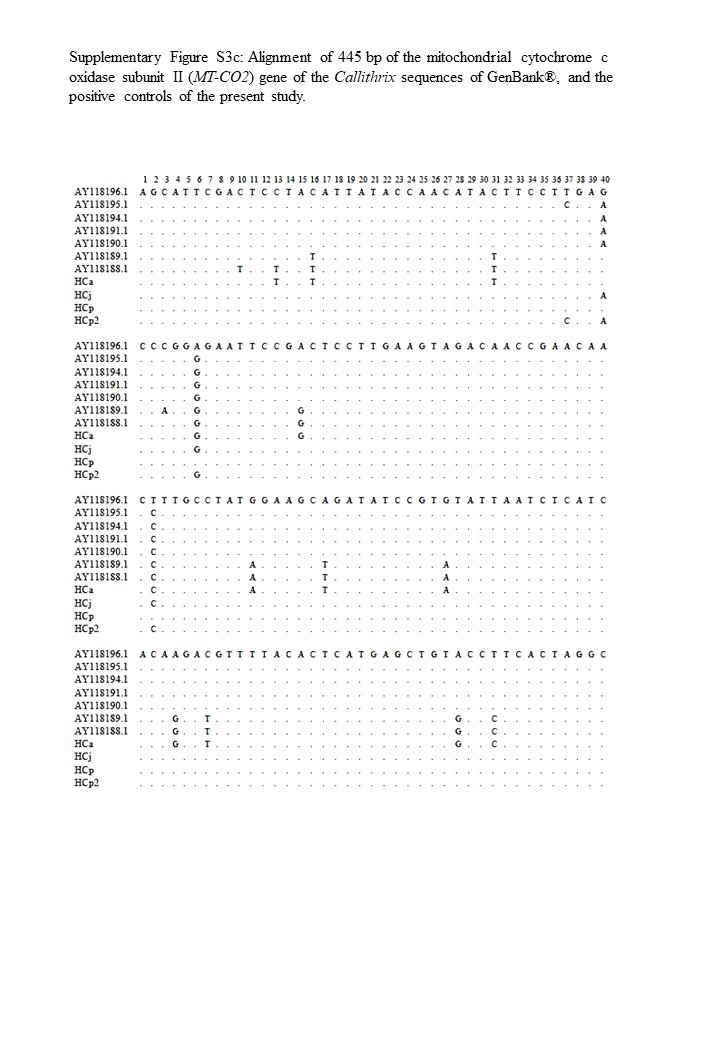

Supplement: Supplementary file 5 — Supplementary Figure S3c. [file 41598_2021_4276_MOESM5_ESM.jpg]

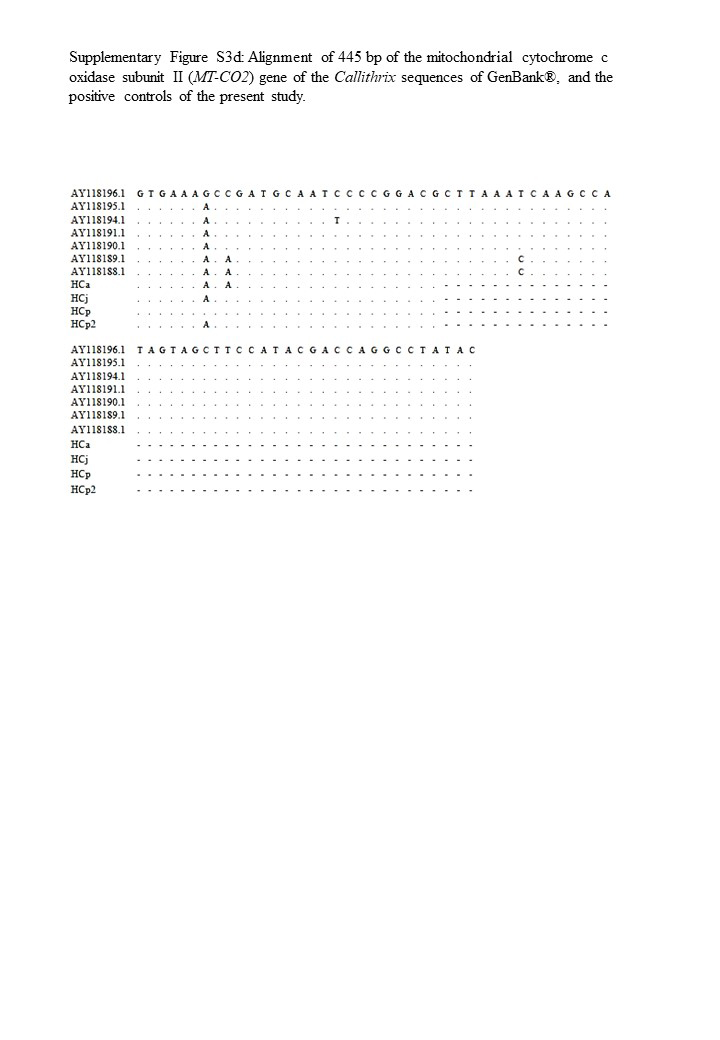

Supplement: Supplementary file 6 — Supplementary Figure S3d. [file 41598_2021_4276_MOESM6_ESM.jpg]

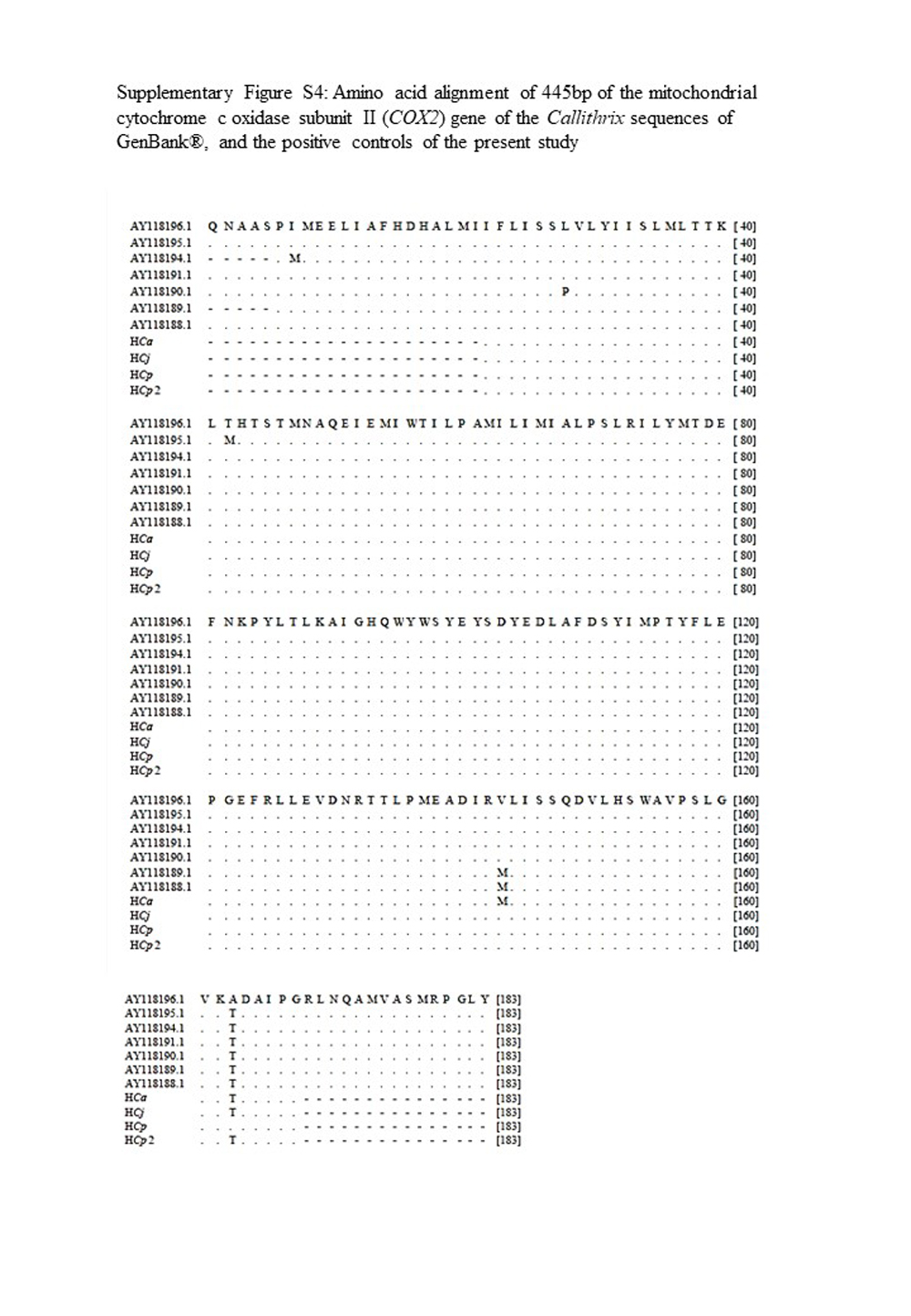

Supplement: Supplementary file 7 — Supplementary Figure S4. [file 41598_2021_4276_MOESM7_ESM.jpg]

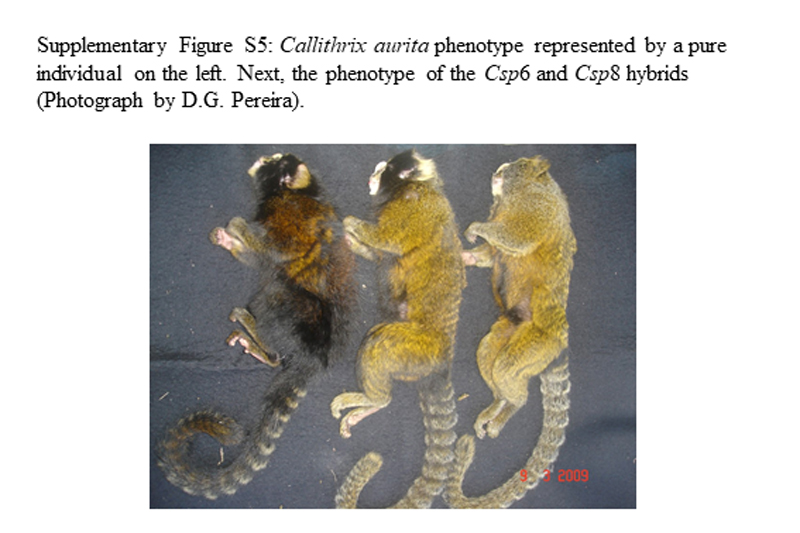

Supplement: Supplementary file 8 — Supplementary Figure S5. [file 41598_2021_4276_MOESM8_ESM.jpg]
