# Supplementary material for: Uniparental genetic markers to investigate hybridization in wild-born marmosets with a mixed phenotype among Callithrix aurita and invasive species
Source: Sci Rep. 2022 Jan 27;12:1487. doi: 10.1038/s41598-021-04276-7 (PMC8795268; doi:10.1038/s41598-021-04276-7)
Supplement: Supplementary file 9 — Supplementary Table S1. [file 41598_2021_4276_MOESM9_ESM.docx]

Supplementary Table S1: Thirty-seven parsimony informative sites in 455 base pair of the mitochondrial cytochrome c oxidase subunit II (*COX2*) gene of *Callithix aurita* (H*Ca*), *C. jacchus* (H*Cj* and *Callitrix penicillata* (H*Cp*1, 2 and 3) haplotypes. Diagnostic sites are marked*.

| Nucleotide Site | H*Ca* | H*Cj* | H*Cp*1 | H*Cp*2 | H*Cp*3 |
| --- | --- | --- | --- | --- | --- |
| 2 | C | T | C | C | T |
| 5 | T* | C | C | C | C |
| 9 | T | C* | T | T | T |
| 32 | T | C | T | C | C |
| 38 | T* | C | C | C | C |
| 56 | A | A | A | G* | A |
| 64 | C | C | C | T* | C |
| 80 | A* | G | G | G | G |
| 101 | A* | G | G | G | G |
| 110 | C* | T | T | T | T |
| 134 | T | T | T | C* | C |
| 158 | T* | C | C | C | C |
| 194 | C* | T | T | T | T |
| 197 | T | A | A | A | T |
| 200 | T* | C | C | C | C |
| 212 | T* | C | C | C | C |
| 224 | A | A | G* | A | A |
| 233 | C | T | C | T | C |
| 269 | T* | C | C | C | C |
| 272 | T* | C | C | C | C |
| 275 | T* | C | C | C | C |
| 290 | T* | C | C | C | C |
| 296 | T | T | T | C* | T |
| 299 | G | A | A | A | G |
| 305 | G | G | G | G | A* |
| 314 | G* | A | A | A | A |
| 341 | C | C | C | C | T* |
| 350 | A* | G | G | G | G |
| 356 | T* | A | A | A | A |
| 366 | A* | G | G | G | G |
| 383 | G* | A | A | A | A |
| 386 | T* | C | C | C | C |
| 407 | G* | A | A | A | A |
| 410 | C* | T | T | T | T |
| 426 | A | A | A | A | G* |
| 428 | A* | C | C | C | C |
| 437 | C | C | T* | C | C |
